# Residential construction cost: An Italian survey

**DOI:** 10.1016/j.dib.2017.02.005

**Published:** 2017-02-09

**Authors:** Rubina Canesi, Giuliano Marella

**Affiliations:** ICEA - Civil, Environmental and Architectural Engineering - University of Padova, Italy

**Keywords:** Construction cost, Real estate, Education level, Survey, Italy

## Abstract

This paper reports data describing development projects for new buildings according to construction costs in North-East Italy. A survey was carried out on local companies undertaking new residential development projects in two Italian regions (Veneto and Lombardy). The aim of this survey was to record new real estate construction projects, collecting both technical and socio-economic cost features. It is extremely difficult to collect such data for the Italian real estate construction sector, due to its lack of transparency, so that the novelty for the Italian scenario is the dataset itself. Another interest perspective of this survey is that socio-economic characteristics were also recorded; they are often studied in urban economics, but are usually related to property purchase prices and values, not to construction costs. The data come from an analysis of Canesi and Marella regarding the relationship between the trend of construction costs and the socio-economic conditions of the reference setting, such as the mean years of schooling of the workforce, housing market trends, and average per capita income.

**Specifications Table**

**Table:** 

Subject area	Economics
More specific subject area	Real Estate
Type of data	Table
How data was acquired	Survey
Data format	Raw
Experimental factors	Sample pretreatment: observations with incomplete data have been rejected and the survey was limited in only two Italian regions. The surveyed variables have been measured both in nominal and in ordinal scales.
Experimental features	We conduct the survey both by interviews and questionnaires; examining both technical and socio-economic features of the development project. Records try to reflect the socio-economic characteristic of the location the project take place.
Data source location	Regions: Veneto and Lombardia; Italy.
Data accessibility	Data are with this article

**Value of the data**

• The data partially fill the gap in information on Italian real estate construction costs, due to problems in collecting it, since the Italian scenario is highly opaque and not systematically recorded.
• The records describe new development projects surveying not only technical but also socio-economic features, a novelty on international level.
• The raw data are easy to interpret and can be processed by qualitative and quantitative statistical analysis, e.g., rough set analysis and hedonic regression models.
• The data can identify the impact of some socio-economic and geographic variables on the unitary construction costs of new real estate development projects.
• Family incomes and construction costs are recognized as influential factors in calculating real estate values [1], [2], [3], but there are very few data and studies attempting to identify the relationships between construction costs and socio-economic features.

## 1 Data

First, we must emphasize that the Italian real estate market is highly opaque and that Italian construction companies hardly reveals information about their building sites, costs or corporate profiles. Therefore, more than elsewhere on the international scene, it is very difficult to collect data, which are private and not publicly recorded or cataloged [4], [5]. In the Italian literature, datasets have little data and the related studies analyze on average 70–80 property [6]. Therefore, our dataset contains information on 70 new residential development projects in North-East Italy, presented between 2006 and 2015. Table 1 lists the surveyed variables (selected both by consulting literature and according to the purposes of this survey), identified by a coding system and clustered into four groups, it also defines their measurement scales, as theorized by Stevens [7].

## 2 Experimental design, materials and methods

Being aware of the low number of cases available, we restricted our analysis and sample new builds in a limited area, rather than the whole country; in order to remove several variables relating to purely territorial dynamics. We establish some basic characteristics shared by all the selected projects, thus ensuring some degree of homogeneity. These homogeneous characteristics are: type of construction (residential apartment block); type of development (new build); period of construction (2006–2015); localization in Veneto and Lombardy regions.

We submitted a questionnaire (Table 2) to several qualified operators in the building sector active in the reference area, stakeholders working for medium-sized enterprises. They were asked to complete a chart, so that we could sample various types of development projects.

After collecting all the survey charts, we compiled the proposed dataset, summarizing and processing its characteristics. The variables selected included some chosen from those examined in the literature [8], [9], plus several others judged to be better at interpreting the socio-economic characteristics and formal education level of the local populations, processed by referring to the localization of the building project.

The first category, *Building Characteristics*, describes the project in physical and technical terms. We chose five variables that intrinsically represent the physical characteristics of an apartment block. The Volume (Vol) of the building in m^3^, including all interior volumes above ground plus 60% of those below ground; Number of Storeys (NS), to survey possible relationships existing between the height and density of a building and its construction cost per m^3^; Quality (Qu) of the building, including design work and materials employed, and whether it was a social housing project or an ordinary development (SHO). Lastly, we surveyed the Construction cost (CC, €/m^3^). All the CC were net of planning fees, which were also surveyed with the submitted questionnaires. Furthermore, the CC are update by applying the *ISTAT index of residential construction cost* to the whole sample, to avoid any temporal bias [10].

*Development Characteristics* describe the timing necessary to complete the process and the developer profile. First was Duration of the building site (Du), in months; and the Size of the construction Company (CS). All consulted companies were classified by European legislation as medium-sized enterprises [11]; as they employ fewer than 250 persons, their annual turnover does not exceed EUR 50 million, and their annual balance sheet total not exceeding EUR 43 million. We therefore clustered this variable in an ordinal scale from 1 to 3, in which 1=companies with an annual turnover of 10–20 million euro; 2=those with an annual turnover of 20–35 million euro, and 3, those with an annual turnover of 35–50 million euro.

The category *Real Estate Market Characteristics* is represented by two variables. The first is the net Surface Planned to be built (SP) in the municipality in question, which represents the vitality of the property market in a given area and is calculated in m^2^ for each province surveyed. The second variable is the unit market Value (Val, in €/m^2^) for each province over the construction time, deduced from market prices quoted in the database of the *Consulente Immobiliare* [12].

In order to capture socio-economic characteristics from the surveyed locations, we identified two main variables: mean gross Income (Inc) of the population in the reference municipality (in €); and formal education, referring to the Number of university Graduates (NGr) in the population of the municipality where the property is to be built.

The data are useful in examining possible relationships existing between residential construction cost and socio-economic features. Such relationships are predicted to be positive, interpreting the literature related to real estate market value, although possible correlations with construction costs have not been demonstrated yet [13], [14].

## Funding

This research did not receive any specific grant from funding agencies in the public, commercial, or not-for-profit sectors.

## Figures and Tables

**Table 1:** Surveyed variables.

**Cluster**	**Code**	**Variable**	**Measur. scale**	***Coding system***
*Building Characteristics*	CC	Construction Cost	Ratio	€/m^3^
	Vol	Volume	Ratio	cubic meter (m^3^)
	NS	Number of Storeys	Interval	n°
	SH/OD	Social Housing or not	Dummy	0: social; 1: normal develop
	Qu	Material finishing	Ordinal	1: poor, 2: adequate, 3: fairly good, 4: good, 5: excellent
*Development Ch.*	Du	Duration of construction	Interval	Number of months
	CS	Company Size	Ordinal	1: low; 2: medium; 3: high annual turnover
*Real Estate Market Ch.*	SP	Net surface planned to be built	Ratio	Square meter (m^2^)
	Val	Market Value	Ratio	€/m2
*Socio-economic Ch.*	NGr	Numbers of graduates	Interval	n°
	Inc	Incomes	Ratio	€/year inhabitant

**Table 2:** Survey chart.

**Surveyed characteristics**	**Unit of measure/classification/description**
City, province	
Social housing or ordinary development	0/1
Urbanized or unurbanized soil	0/1
Planning fees	€
Building volume	m^3^
N° of storeys	(0-*n*)
Quality of finishing	0–5
Building shape	
Plant design	
Starting date of construction	dd/mm/yy
End of construction	dd/mm/yy
Number of employees	(0-*n*)
Annual turnover	
Cost of construction	€
